# Characterization of six microsatellite loci in *Myrica faya* (Myricaceae) and cross amplification in the endangered endemic *M. rivas-martinezii* in Canary Islands, Spain

**DOI:** 10.1590/S1415-47572009005000006

**Published:** 2009-01-10

**Authors:** Miguel A. González-Pérez, Craig Newton, Pedro A. Sosa, Elizabeth Rivero, Edna A. González-González

**Affiliations:** 1 Departamento de Biología, Universidad de Las Palmas de Gran Canaria, Canary IslandsSpain; 2 ATG Genetics Inc, Vancouver, British ColumbiaCanada

**Keywords:** * Myrica rivas-martinezii*, *Myrica faya*, microsatellite, Canary Islands, genetic diversity

## Abstract

Six novel polymorphic microsatellite markers were isolated from enriched libraries in *Myrica faya* Ait., recently renamed *Morella faya*, (fayatree, firetree, or firebush) in order to examine the genetic diversity in natural populations. Also, test cross-specific amplification and genetic diversity in *Myrica rivas-martinezii,* which is endemic on the Canary islands. Microsatellite loci were screened in 225 individuals of both species from different islands of the Canarian archipelago. All markers were successfully amplified from both *Myrica* species, with an average number of 6.5 and 9.3 alleles per locus in *M. rivas-martinezii* and *M. faya*, respectively. There was no evidence for linkage disequilibrium between loci, and the probability of null alleles ranged from 0.01 to 0.17.

*M. faya* is native to the northern islands of Macaronesia, the Azores, Madeira, and the Canaries in the North Atlantic Ocean. It is found in most laurel forest plots, but also grows well at degraded sites and outside the range of the laurel forest ecosystems. *M. faya* is quite abundant due to its high colonizing capacity (Bañares *et al.*, 2004). In addition, *Myrica faya* has been recognized as one of the twelve most noxious plants alien to Hawaii due to its ability to rapidly and aggressively invade and colonize the Hawaiian environment (Smith, 1985).

*M. faya* co-occurs very close (separated by few meters) to *Myrica rivas-martinezii* A. Santos. The latter is an endangered endemic species of the laurel forests on the Canary Islands. *M. rivas-martinezii* is found on rather poor soils along the inferior margins of the laurel forest domain, especially in isolated locations that have been the object of multiple exploitations. It is a perennial, woody species that was first time described in El Hierro in 1980 (Santos-Guerra, 1980). This dioecious tree species occurs on only three islands (El Hierro, La Gomera and La Palma) of the Canarian archipelago. Its most abundant population (with about 40 individuals) is located at El Fayal, in an area of approximately 90 km^2^ on El Hierro Island. Only ten isolated individuals are known in La Gomera Island, six of which have male flowers, all in different locations and separated by linear distances ranging from 1 to 7 km, but isolated by deep ravines and cliffs. In La Palma, the only two known individuals (one male and one female) are separated by more than 20 km (Bañares *et al.*, 2004). *Myrica rivas-martinezii* has been classified as Critically Endangered according to IUCN categories (VVAA, 2000). It is also protected by the Canarian Government (Boletín Oficial de Canarias – BOC, 2001) and the European Habitat Directive (Beltrán *et al.*, 1999).

Previous studies showed no evidence for genetic differentiation on the basis of the sequences of either the pastid rbcl gene, which encodes the large subunit of rubisco, or the 18S-26S nuclear internal transcribed spacer (ITS) region (Huguet *et al.*, 2005). Yet, Batista *et al.* (2004) showed higher genetic diversity levels in *M. rivas-martinezii* using RAPDs.

We sampled 41 specimens of *Myrica rivas-martinezii* and 183 of *M. faya* in El Hierro, La Gomera, and La Palma Island (Table 1). Microsatellite loci were isolated from *M. faya* genomic DNA using biotin/streptavidin enrichment (Khasa *et al.*, 2000). Briefly, genomic DNA was digested with restriction endonucleases (*Hae*III, *Alu*I and *Rsa*I) and linkers were added using T4 DNA ligase. Linker adapted total genomic fragments were then enriched by hybridization with 5' biotin AC_12_ and TC_12_ followed by separation with streptavidin paramagnetic beads (M270S, Invitrogen, CA). After amplification with linker-specific primers, enriched fragments were cloned into plasmid vectors (pGEM3Z+, Promega) and single colonies containing ACn or TCn microsatellites were identified by colony hybridisation. After one round of biotin enrichment, 75/1000 ampicillin resistant colonies contained microsatellite sequences. Inserts from 23 positive colonies were amplified with M13 universal forward and reverse primers, treated with exonuclease I and Antarctic alkaline phosphatase (New England Biolabs) , and then sequenced from both orientations using a ABI3730 capillary electrophoresis system (NAPS Service, University of British Columbia).

PCR primers complementary to the flanking regions of 13 loci were designed with approximately 40% GC and avoiding palindromic sequence motifs. Out of 13 microsatellite loci analyzed, six yielded PCR products of expected size and were considered robust and predictable enough for further analyses (Table 2).

Total DNA was extracted following the Dellaporta *et al.* (1983) method modified by Corniquel and Mercier (1994). Each 25 μL PCR reaction contained approximately 20 ng DNA, 10 pmol of each primer, 0.25 μL BSA (0.4%), as well as PCR Master Mix (Reddy-Mix, ABgene, Surrey, UK). Amplifications were carried out using the following conditions: 3 min denaturation at 95 °C, 35 cycles of 30 s denaturation at 95 °C, 30 s annealing at 55 °C, and 1.5 min elongation at 72 °C, followed by 5 min final elongation at 72 °C. The products were detected using an ABI 3100 Genetic Analyzer and fragment sizes were determined using GENESCAN v. 2.02 and GENOTYPER v. 1.1 (Applied Biosystems, Inc.). We identified allele peak profiles at each locus and assigned a genotype to each individual.

Exact Hardy-Weinberg tests to measure the significance of deviations from the null hypothesis of random union of gametes (Guo and Thompson, 1992) were carried out on natural populations of *M. rivas-martinezii* and *M. faya* using Fisher exact test, both for each pair of loci and within each population using GENEPOP 1.2. (Raymond and Rousset, 1995). Basic genetic diversity indices, mean number of alleles (*A*), the observed (*Ho*), and unbiased expected (*He*) heterozygosities (Nei, 1978), paternity exclusion probability (*Q*) (Weir, 1996), and probability of genetic identity (*I*) (Paetkau *et al.*, 1995), were estimated using IDENTITY 1.0. The combined probability of paternity exclusion, *QC* = 1 - [Π(1 – *Q*_*i*_)] and the combined probability of genetic identity *IC* = Π*I*_i_ were also estimated for overall loci. The proportion of null alleles for each locus was calculated as (*He*-*Ho*)/1 +He, following Brookfield (1996).

All six loci were polymorphic, the number of alleles ranging from 3 (M24) to 16 (M10), while *Ho* ranged from 0.30 (M5) to 0.88 (M10), and gene diversity (*He*) from 0.32 (M5) to 0.89 (M10). Only one locus (M24) showed values of proportion of null alleles over 10% in both species, suggesting the possibility of null alleles (Table 2). Except for locus M24, none of the other loci showed significant deviations from Hardy-Weinberg equilibrium in *M. rivas-martinezii*, (data not shown). The M24 locus was estimated to have a null allele frequency of 0.16.

While there was no evidence for linkage disequilibrium between loci in the data set of 225 individuals of both *Myrica* species, three (M11, M20 and M24) of the six analyzed loci showed significant defect of heterozygote in *M. faya*. These three loci were estimated to have null allele frequencies of 0.10, 0.04, and 0.17, respectively. Many of the loci showed high probability of paternity exclusion, which should provide powerful markers for paternity exclusion when genotype data from several loci are combined. Total paternity exclusion probability (*QC*) for this set of six microsatellite loci was estimated at 0.981 for *M. faya*, and 0.941 for *M. rivas-martinezii*. Total probabilities of identity (*IC*) were 5.71 x 10^-6^ and 9.03 x 10^-5^ for *M. faya* and *M. rivas-martinezii*, respectively.

Genetic diversity recorded for the endemic species *M. rivas-martinezii* was similar to those described for other endangered endemic species of the Canary Islands, for example *Bencomia exstipulata* (*A* = 6.20, *Ho* = 0.43, *He* = 0.65) (González-Pérez *et a*l., 2004). In contrast genetic variability in *M. faya* was higher than corresponding values detected in other, not endangered native species of the Canary Islands, *e.g.*, *B. caudata* (*A* = 6.40, *Ho* = 0.47, *He* = 0.62) (González-Pérez *et al.*, 2004). This may reflect the high colonizing capacity of *M. faya* (Bañares *et al.*, 2004).

Altogether, the primers described in this paper can provide useful markers to investigate genetic relationship between *Myrica rivas-martinezii* and *M. faya*.

## Figures and Tables

**Table 1:** *Myrica rivas-martinezii* and *M. faya* populations analysed in the Canary Islands.

Island	Species	*N*
La Gomera	*M. rivas-martinezii*	10
	*M. faya*	132

El Hierro	*M. rivas-martinezii*	30
	*M. faya*	38

La Palma	*M. rivas-martinezii*	2
	*M. faya*	13

Total	*M. rivas-martinezii*	42
	*M. faya*	183

*N* = sample size.

**Table 2:** Characteristics of six microsatellite loci developed for *Myrica faya* and cross amplification in *M. rivas-martinezii*. Number of alleles (*A*), observed heterozygosity (*Ho*), expected heterozygosity (*He*), probability of paternity exclusion (*Q*), probability of genetic identity (*I*), proportion of null alleles (*Pna*), and GenBank accession number.

Locus	Species	Repeat motif	Size range (bp)	*A*	*Ho*	*He*	*Pna*	*Q*	*I*	PCR Primer sequence (5'→ 3')	GenBank accession
M5	*M. faya*	(GA)_21_	197-228	7	0.56	0.59	0.02	0.31	0.25	F:6FAM-GCCATCTGCATACCACAACAG	AM922310
	*M. rivas-martinezii*			5	0.31	0.32	0.01	0.16	0.49	R:AATGGGGAGAGGGTATCTAG	

M10	*M. faya*	(AG)_24_	155-191	16	0.88	0.89	0.01	0.78	0.02	F:VIC-TGCTTATTTCTTTGACACGACC	AM922311
	*M. rivas-martinezii*			13	0.72	0.80	0.06	0.62	0.06	R:ATGTCATGTGTGATTTTTCTCAAA	

M11	*M. faya*	(CT)_27_	169-193	11	0.50	0.67	0.10	0.42	0.17	F:NED-GCCTTCAGATCAAAGTATGCA	AM922312
	*M. rivas-martinezii*			6	0.51	0.58	0.04	0.36	0.22	R:ACCAAGTTGATGATCATGTGTCA	

M18	*M. faya*	(TC)_20_	315-343	12	0.73	0.76	0.01	0.54	0.10	F:6FAM-ATGATATGTAGTGAAAGAGAGCAG	AM922313
	*M. rivas-martinezii*			8	0.76	0.72	0.01	0.47	0.13	R:ATGCATAAAACTAGCCCTTAC	

M20	*M. faya*	(GT)_18_	166-181	6	0.46	0.53	0.04	0.30	0.27	F:VIC-GTGCATCCTAACTACGGAGG	AM922314
	*M. rivas-martinezii*			4	0.36	0.37	0.01	0.20	0.42	R:CTTGTAGCCACCCCAATATGG	

M24	*M. faya*	(GA)_20_	156-162	4	0.31	0.59	0.17	0.32	0.24	F:NED-CCTCCGTTGTGCATAGTCGTC	AM922315
	*M. rivas-martinezii*			3	0.30	0.58	0.16	0.31	0.24	R:GAATTCTTCGACCGGAACTTCC	

Mean^ ^	*M. faya*^ ^			9.3^ ^	0.57^ ^	0.67^ ^	0.06^ ^	*QC* = 0.981; *IC* = 5.71110^-6^			
	*M. rivas-martinezii*^ ^			6.5^ ^	0.49^ ^	0.56^ ^	0.05^ ^	*QC* = 0.941; *IC* = 9.02610^-5^			
